# Treatment and Follow-up Care Associated With Patient-Scheduled Primary Care Telemedicine and In-Person Visits in a Large Integrated Health System

**DOI:** 10.1001/jamanetworkopen.2021.32793

**Published:** 2021-11-16

**Authors:** Mary Reed, Jie Huang, Ilana Graetz, Emilie Muelly, Andrea Millman, Catherine Lee

**Affiliations:** 1 Kaiser Permanente Division of Research, Oakland, California; 2 Rollins School of Public Health, Emory University, Atlanta, Georgia; 3 Department of Internal Medicine, Kaiser Permanente Santa Clara, Santa Clara, California

## Abstract

**Importance:**

Telemedicine visits can offer patients convenient access to a clinician, but it is unclear whether treatment differs from that with in-person visits or how often patients require in-person follow-up.

**Objective:**

To examine whether physician prescribing and orders differ between telemedicine and office visits, whether physicians conducting telemedicine visits are more likely to require in-person follow-up, and whether telemedicine visits are associated with more health events.

**Design, Setting, and Participants:**

This cohort study included all patients who scheduled primary care appointments through the patient portal of a large integrated health care delivery system newly implementing patient-scheduled video telemedicine visits from January 2016 to May 2018.

**Main Outcomes and Measures:**

Adjusted rates of any medication prescribed or laboratory tests or imaging ordered and rates of follow-up health care utilization (in-person visits, emergency department visits, and hospitalizations) within 7 days after the index visit, stratified by index primary care visit type, were generated using multivariable adjustment for patient, access, and clinical characteristics.

**Results:**

This study included 1 131 722 patients (611 821 [54%] female; mean [SD] age, 43 [22] years) with 2 178 440 total appointments (307 888 [14%] telemedicine), of which 13.5% were for patients younger than 18 years, 22.2% were for patients 65 years or older, and 54.9% were for female patients. After adjustment, 38.6% (95% CI, 38.0%-39.3%) of video visits, 34.7% (95% CI, 34.5%-34.9%) of telephone visits, and 51.9% (95% CI, 51.8%-52.0%) of office visits had any medication prescribed; laboratory tests or imaging were ordered for 29.2% (95% CI, 28.5%-29.8%) of video visits, 27.3% (95% CI, 27.1%-27.5%) of telephone visits, and 59.3% (95% CI, 59.3%-59.4%) of clinic visits. After adjustment, follow-up visits within 7 days occurred after 25.4% (95% CI, 24.7%-26.0%) of video visits, 26.0% (95% CI, 25.9%-26.2%) of telephone visits, and 24.5% (95% CI, 24.5%-24.6%) of office visits. Adjusted emergency department visits and rates of hospitalizations were not statistically significantly different by primary care index visit type.

**Conclusions and Relevance:**

In this cohort study of patient self-scheduled primary care telemedicine visits within ongoing patient-physician relationships, prescribing and orders were significantly lower for telemedicine visits than for clinic visits, with slightly higher follow-up office visits for telemedicine but no difference in health events (emergency department visits or hospitalizations). Video or telephone visits may be a convenient and efficient way to access primary care and address patient needs.

## Key Points

**Question:**

In patient-scheduled primary care video or telephone visits with existing physicians, do rates of treatment or follow-up office or emergency visits differ from rates of in-person visits?

**Findings:**

In this cohort study of 1 131 722 patients, adjusted rates of prescribing and nonmedication orders were significantly lower for telemedicine visits than for clinic visits, with slightly higher rates of follow-up office visits after telemedicine visits but no significant difference in rates of 7-day emergency visits or hospitalizations.

**Meaning:**

The findings suggest that video or telephone visits may offer a convenient way to address some primary care needs within ongoing patient-physician relationships, without substantially higher rates of follow-up office visits or health events (emergency department visits or hospitalizations).

## Teaser

This cohort study evaluates whether rates of treatment or follow-up office or emergency visits associated with telemedicine visits differ from those associated with in-person visits in a large integrated health system.

## Introduction

The COVID-19 pandemic unexpectedly shifted the US health care system toward a large volume of telemedicine care. Longer-term telemedicine use in primary care, however, may not be driven primarily by social distancing efforts but instead may focus on ways that telemedicine can offer a convenient option for expanding access to health care.^1^ Many patients and health care practitioners now recognize that video or telephone telemedicine can offer patients access to a clinician without transportation arrangements, time off from work, or time spent in a waiting-room. Still, it is unclear whether telemedicine visits adequately address the patient’s clinical concern, are more likely to require subsequent follow-up outpatient care, or are more likely to be followed by a serious health event that requires an emergency department visit or hospital stay.

In addition, prior evidence from direct-to-consumer telemedicine, which is often not integrated with the patient’s regular physician or full electronic health record, suggests potential over-prescribing and differences in physician orders and follow-up visits when primary care is delivered through telemedicine compared with through a typical office visit.^2,3,4^ In contrast, little evidence exists about these care processes in telemedicine visits between patients and their own primary care physicians.^5^

In a setting with prepandemic patient-scheduled video and telephone telemedicine with patients’ own primary care physicians, we compared telemedicine visits and in-person office visits with regard to care processes and postvisit health care utilization. We hypothesized that serious outcomes would be rare after telemedicine visits but that some patients would require additional follow-up visits.

## Methods

### Setting

In this cohort study, we examined primary care visits in a large integrated health care delivery system with more than 4 million members, Kaiser Permanente Northern California, which uses a comprehensive outpatient-inpatient electronic health record (EHR; including outpatient, emergency, inpatient, laboratory, imaging, and pharmacy history) and patient portal. The institutional review board of the Kaiser Foundation Research Institute approved the study protocol and materials and waived the requirement for written informed consent for participants because the study used deidentified data. This study followed Strengthening the Reporting of Observational Studies in Epidemiology (STROBE) reporting guideline.

All clinicians in the setting have had access to video visit technology since 2014, and since 2016, patients scheduling a primary care appointment through the patient portal have been required choose their visit type: office, video, or telephone visit. The only exception was for visits designated as a “routine physical,” which were offered only as office visits. Available clinicians included patients’ own personal primary care clinician (primarily MDs, including nurse practitioners) or other primary care clinicians the patient had visited recently. The scheduling availability and clinicians available were comparable across visit types, with appointment availability generally within 3 days (often available the same day). Further details about video visits used in this setting, patient characteristics associated with use, and patient experiences have been described in prior publications.^6,7,8^

As in an office visit, clinicians conducting telemedicine visits had full access to the patient’s EHR history and documented telemedicine visits directly within the EHR. Patients could receive a call for a telephone visit at any phone number and could access video visits from home or elsewhere in their daily lives directly through any internet-connected and video-enabled computer or mobile device.

### Study Population

We studied all completed primary care appointments booked via the patient portal from January 2016 to May 2018. We included only index visits without any other clinical visits within the previous 7 days to define a relatively distinct patient-initiated care-seeking episode. We also excluded visits for routine physical, which were not eligible for telemedicine.^6^ We also identified subsets of visits with diagnoses of upper respiratory tract infection (URI) and skin conditions, 2 commonly reported categories of telemedicine chief complaints and diagnoses.

### Outcome Measures

For each visit, we identified any prescribing or other orders (eg, for laboratory testing or imaging) associated with the visit, including the subset of prescriptions specifically for antibiotics. To characterize short-term follow-up health care utilization, we extracted all office-visits, emergency department visits, and hospitalizations within 7 days after the index primary care visit. We examined each outcome in the full sample of all patient visits, and in the subset of visits with diagnoses of URI and skin conditions because these were common visit reasons with prior telemedicine evidence.^9^

### Covariates

When comparing outcomes associated with index visit type, we accounted for a broad set of visit covariates using several types of patient characteristics previously shown to be associated with visit-type choice, including patient sociodemographic characteristics, and other measures of accessibility and affordability grouped into technology access, in-person visit barriers, and patient-clinician affiliation.^6^ We used the EHR to identify patient sociodemographic characteristics (age, sex, race and ethnicity, and language preference), patient’s residential address to define patient neighborhood socioeconomic status (2010 US census measures at the census block group level), and neighborhood residential high-speed internet access level (Federal Communications Commission census tract level data). We included patients’ mobile portal use in the prior 365 days as a measure of mobile device access and video visit use in the prior 365 days as a measure of video visit experience. We extracted the patient’s insurance benefit cost-sharing for office visits. We also extracted the estimated drive time from the patient’s residence to the nearest health system medical facility and the type of parking offered at that facility (free parking lot or structure vs paid parking structure). We used automated data to identify whether the index visit was scheduled by a family care partner with permissions to act for the patient through the patient portal and whether the appointment was scheduled with the patient’s own personal primary care clinician.

### Statistical Analysis

We used a multivariable logistic regression model to examine the association between index visit type (in-person visit, video visit, or telephone visit) and outcomes, including any prescribing, antibiotic prescribing, and orders during the visit; any in-person visit, emergency department visit. and hospitalization within 7 days after the index visit. The model was adjusted for patient age, sex, and race and ethnicity; neighborhood socioeconomic status; preferred language for health care; out-of-pocket cost-sharing for office visits; drive time to clinic; facility parking garage and fee; neighborhood internet access level; mobile portal use in prior 365 days; video visits in prior 365 days; whether the appointment was booked by a care partner on behalf of the patient; whether the clinician was the patient’s own personal primary care clinician; patient medical problem (*International Statistical Classification of Diseases and Related Health Problems, Tenth Revision* code grouping of primary diagnosis); whether the patient had preexisting chronic conditions (in clinical registry of asthma, heart failure, diabetes, and hypertension in quarter prior to index visit); and medical center. We examined each outcome using a separate logistic regression model because standard composite measures of study outcomes were not readily available and each outcome represents a clinically distinct action and outcome. Standard errors were adjusted for repeated visits by patients. For easier interpretation, we calculated an adjusted rate for each outcome from the multivariable logistic regression model as if every visit in the entire cohort was an in-person visit, as if every visit in the entire cohort was a video visit, and as if every visit in the entire cohort was a telephone visit. We repeated the analyses for the 2 subsets of visits with diagnoses of URI and skin conditions (eAppendix in the Supplement). All analyses were conducted using 2-sided tests for significance and with *P* < .05 as the threshold for significance. Analyses were performed using Stata, version 14.2 (StataCorp LLC).

## Results

Among all 2 178 440 patient-scheduled primary care visits scheduled by 1 131 722 patients (611 821 [54%] female; mean [SD] age, 43 [22] years), 1 870 552 (86%) were scheduled as office visits and 307 888 (14%) as telemedicine visits, with 20 115 (7%) of the telemedicine visits by video. Overall, 13.5% of visits were for patients younger than 18 years and 22.2% were among patients 65 years or older; 54.9% were for female patients, 59.8% were for White patients, and 95.1% were for English-speaking individuals (Table).^6^

**Table.** Patient-Scheduled Primary Care Visits by Visit Type

Characteristic	Visits, No. (%)			
	Total (N = 2 178 440)	Video visit (n = 20 115)	Telephone visit (n = 287 773)	Office visit (n = 1 870 552)
Age, y				
<18	294 021 (13.5)	3608 (17.9)	24 357 (8.5)	266 056 (14.2)
18 to <45	682 955 (31.4)	9989 (49.7)	114 427 (39.8)	558 539 (29.9)
45 to <65	717 404 (32.9)	5370 (26.7)	99 675 (34.6)	612 359 (32.7)
≥65	484 060 (22.2)	1148 (5.7)	49 314 (17.1)	433 598 (23.2)
Sex				
Female	1 195 097 (54.9)	11 423 (56.8)	175 324 (60.9)	1 008 350 (53.9)
Male	983 343 (45.1)	8692 (43.2)	112 449 (39.1)	862 202 (46.1)
Race and ethnicity				
Asian	448 238 (20.6)	6076 (30.2)	56 200 (19.5)	385 962 (20.6)
Black	112 482 (5.2)	1390 (6.9)	19 017 (6.6)	92 075 (4.9)
Hispanic	288 143 (13.2)	2450 (12.2)	39 803 (13.8)	245 890 (13.2)
White	1 301 808 (59.8)	9906 (49.3)	168 741 (58.6)	1 123 161 (60.0)
Other	27 769 (1.3)	293 (1.5)	4012 (1.4)	23 464 (1.3)
Low neighborhood SES	339 296 (15.6)	3059 (15.2)	47 766 (16.6)	288 471 (15.4)
English-language speaker	2 071 074 (95.1)	19 246 (95.7)	275 281 (95.7)	1 776 547 (95.0)
Chronic condition				
Any	675 781 (31.0)	3190 (15.9)	88 089 (30.6)	584 502 (31.3)
URI related	222 885 (10.2)	1923 (9.6)	34 152 (11.9)	186 810 (10.0)
Skin related	101 331 (4.7)	2771 (13.8)	8589 (3.0)	89 971 (4.8)

Abbreviations: SES, socioeconomic status; URI, upper respiratory tract infection.

### Prescribing and Ordering

**Figure 1.**
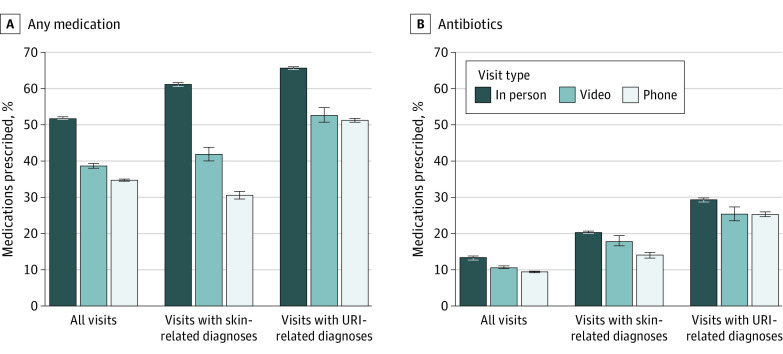
Adjusted Rate of Medication Prescribing by Index Visit Type Adjusted rates of primary care visits with any medication prescribing during the index visit based on results from multivariable logistic regression models adjusting for patient demographics, potential barriers to in-person visits, chronic conditions, *International Statistical Classification of Diseases and Related Health Problems, Tenth Revision* grouping of primary diagnoses, and medical center, with SEs adjusted for repeated visits by patients. Analyses were repeated for subgroups of visits for upper respiratory tract infections (URIs) and skin conditions, 2 commonly reported categories of telemedicine chief complaints and diagnoses. Whiskers indicate 95% CIs.

**Figure 2.**
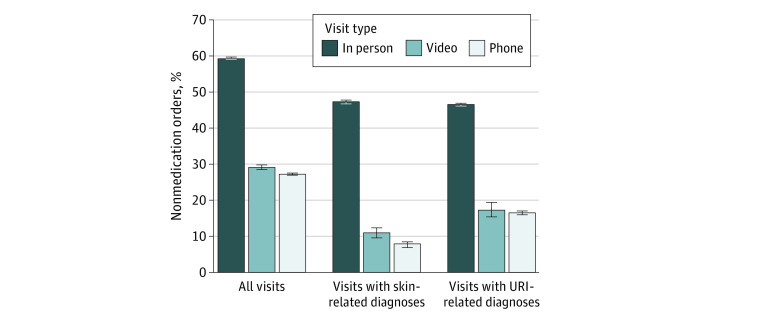
Adjusted Rate of Nonmedication Orders by Visit Type Adjusted rates of primary care visits with any order (eg, laboratory tests or imaging) during the index visit based on results from multivariable logistic regression models adjusting for patient demographics, potential barriers to in-person visits, chronic conditions, *International Statistical Classification of Diseases and Related Health Problems, Tenth Revision* grouping of primary diagnoses, and medical center, with SEs adjusted for repeated visits by patients. Analyses were repeated in subsets of visits for upper respiratory tract infections (URIs) and skin conditions, 2 commonly reported categories of telemedicine chief complaints and diagnoses. Whiskers indicate 95% CIs.

After adjustment (Figure 1 and the eTable in the Supplement), the rate of visits with any medication prescribing was 51.9% (95% CI, 51.8%-52.0%) for office visits, 38.6% (95% CI, 38.0%-39.3%) for video visits, and 34.7% (95% CI, 34.5%-34.9%) for telephone visits; the rate of any antibiotics prescribing was 13.5% (95% CI, 13.4%-13.5%) for office visits, 10.6% (95% CI, 10.2%-11.0%) for video visits, and 9.7% (95% CI, 9.5%-9.8%) for telephone visits. As shown in Figure 2, the adjusted rate of ordering any laboratory test or imaging was 59.3% (95% CI, 59.3%-59.4%) for office visits, 29.2% (95% CI, 28.5%-29.8%) for video visits, and 27.3% (95% CI, 27.1%-27.5%) for telephone visits. This pattern in care processes between office visits and telemedicine visits was consistent among the subset of visits with diagnoses of URI and skin conditions.

### Postvisit Follow-up Events

**Figure 3.**
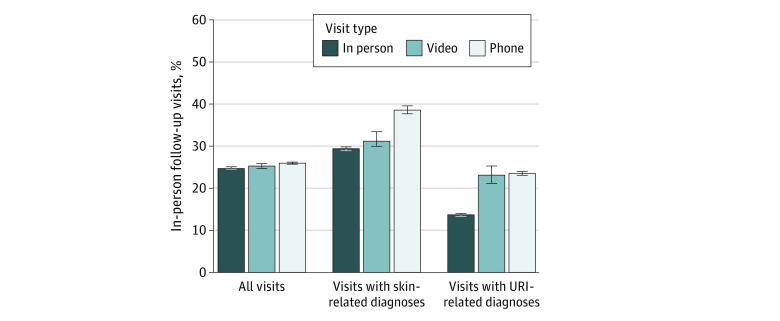
Adjusted Rate of Follow-up Office Visits by Primary Care Index Visit Type Adjusted rates of primary care visits with any in-person office visit within 7 days after the index visit based on results from multivariable logistic regression models adjusting for patient demographics, potential barriers to in-person visits, chronic conditions, *International Statistical Classification of Diseases and Related Health Problems, Tenth Revision* grouping of primary diagnoses, and medical center, with SEs adjusted for repeated visits by patients. Analyses were repeated in subsets of visits for upper respiratory tract infections (URIs) and skin conditions, 2 commonly reported categories of telemedicine chief complaints and diagnoses. Whiskers indicate 95% CIs.

**Figure 4.**
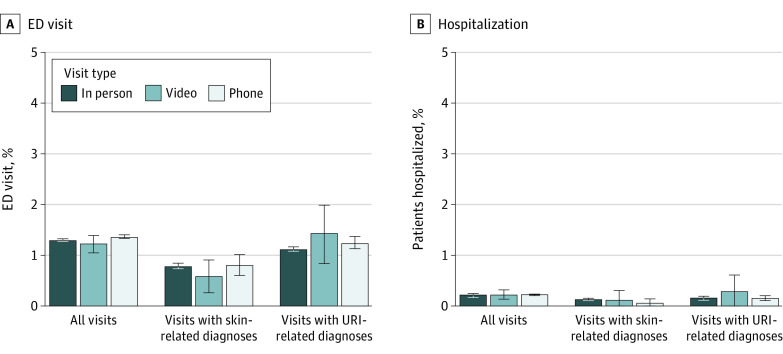
Adjusted Rate of Follow-up Emergency Department (ED) Visits and Hospitalizations by Index Visit Type Adjusted rates of primary care visits with any ED visit or hospitalization within 7 days after the index visit based on results from multivariable logistic regression models adjusting for patient demographics, potential barriers to in-person visits, chronic conditions, *International Statistical Classification of Diseases and Related Health Problems, Tenth Revision* grouping of primary diagnoses, and medical center, with SEs adjusted for the repeated visits by patients. Analyses were repeated in subsets of visits for upper respiratory tract infections (URIs) and skin conditions, 2 commonly reported categories of telemedicine chief complaints and diagnoses. Whiskers indicate 95% CIs.

After adjustment, 24.5% (95% CI, 24.5%-24.6%) of office visits, 25.4% (95% CI, 24.7%-26.0%) of video visits, and 26.0% (95% CI, 25.9%-26.2%) of telephone visits were followed by an in-person office visit in the subsequent 7 days (Figure 3). Adjusted rates of emergency department visits within 7 days were not statistically significantly different between office visits (1.30%, 95%CI, 1.29%-1.32%) and video visits (1.23%; 95% CI, 1.06%-1.40%) but slightly higher among telephone visits (1.37%; 95% CI, 1.33%-1.41%) (Figure 4). Adjusted rates of hospitalizations were not statistically significantly different by primary care index visit type; 0.23% (95% CI, 0.22%-0.24%) of in-person office visits, 0.23% (95% CI, 0.14%-0.32%) of video visits, and 0.22% (95% CI, 0.21%-0.24%) of telephone visits were followed by hospitalization within 7 days.

## Discussion

In a large, integrated health care setting newly offering patient-scheduled primary care telemedicine visits, adjusted rates of prescribing and ordering were statistically significantly lower for telemedicine visits compared with in-person office visits. This was consistent for all visits and for the subset of visits with diagnoses of URI or skin conditions. Return office visit utilization was slightly higher after video and telephone visits than after an in-person primary care visit. Rates of emergency visits and hospitalizations were low and comparable between in-person and video or telephone primary care visits.

We examined the integration of patient-initiated telemedicine visits with ongoing clinical care and patient-clinician relationships in a nonpandemic study period. Although the landscape of telemedicine payment barriers has improved particularly during the COVID-19 pandemic, this study offers an opportunity to examine a study setting with patient-scheduled telemedicine fully integrated into clinical care delivery and into the EHR, with consistent clinicians and availability between visit types. This type of integration between physicians conducting in-person and telemedicine visits and settings is increasingly common under pandemic telemedicine expansions.^10^ In contrast to prior findings from direct-to-consumer telehealth physicians that may not be linked with a patient’s history or ongoing clinician relationships, we found lower rates of antibiotic prescribing via clinically integrated telemedicine visits compared with in-person clinic visits.^2,3^ Our findings of low but differentially higher in-person follow-up visits after telemedicine are consistent with prior findings in direct-to-consumer telemedicine settings.^4^ Still, even in cases in which a telemedicine visit does not serve as a stand-alone visit, there may be additional potential visits in telemedicine in offering initial clinical and site-of-care guidance.

Even as pandemic concerns subside over time and telemedicine use rates continue to evolve, our findings suggest that telemedicine can be used to safely address some concerns when offered in the context of integrated in-person health care services. Aside from social-distancing aspects of telemedicine, patients will likely continue to choose virtual care for convenient health care access, potentially when facing barriers to in-person health care services, such as travel, mobility, taking time off from work, or caregiving responsibilities.^8^ We have previously published associations between patient sociodemographic and technology access characteristics and visit type choice.^6^ In addition, patients may also choose telemedicine if they do not expect that their condition will require in-person examination or services. Despite increasing use and availability of video technology, telephone-based virtual care is often still the dominant type of telemedicine used by patients and physicians, including during the COVID-19 pandemic.^3^ Furthermore, given the potential digital divide in technology access to support video telemedicine visits, our findings of general comparability in adverse health events between telephone and video-based telemedicine visits in primary care suggest that phone visits may continue to offer a simple health care access option without raising safety concerns.^6^

### Limitations

This study has limitations. In this observational study, although our analyses adjusted for a wide range of patient and clinical covariates, we could not rule out unmeasured confounding, especially because the visit type in our study was self-selected by patients. Also, in examining patients self-scheduled visits via the portal, these findings may not represent visits booked by clinic staff at the request of a physician. More research is needed to fully characterize the full range of telemedicine visits used by patients and physicians. Our study outcomes were constructed such that the orders we identified in a given visit (eg, the ordering of future preventive tests) or visits in a given follow-up period (eg, new health problems may arise) may not have been directly related to the index visit. Because the subgroup analyses were limited to 2 specific common clinical subsets (skin and URI concerns), further research is also needed to characterize the use and outcomes associated with the wide range of specific clinical concerns and pathways addressed in primary care. In using a relatively short number of days to define a distinct care-seeking episode, we were not able account for patients’ longer-term history with a condition or the condition’s severity or its effect on the current visit type or outcome. This study did not aim to directly assess clinical appropriateness of a given visit type or health care utilization choice; further research is needed to closely examine this question.

Also, the generalizability of our study is limited to the care delivery and nonpandemic setting of the data. Further research in the use of telemedicine during the COVID-19 pandemic is needed, particularly as the balance between telemedicine and in-person services and between telephone and video visits continues to evolve. Similarly visit types may vary between health care systems and geographic areas and may also vary over time.

## Conclusions

In contrast to direct-to-consumer telemedicine, in this cohort study of patients using telemedicine to visit their own primary care physicians, we did not find evidence of overordering or overprescribing. Future study is needed to further examine the quality and safety of telemedicine within ongoing patient-physician relationships. Patient-scheduled video or telephone visits with their own primary care physicians were associated with a modestly higher rate of follow-up outpatient office visits than were initial in-person visits. This might be expected owing to the lower barrier to entry for telemedicine and to some level of anticipated need for follow-up. There were, however, no statistically significant differences in the rate of emergency department visits or hospitalization after telemedicine. Video or telephone visits may be a convenient and safe way for patients to address some primary care needs without generating a substantial number of follow-up office visits or experiencing health events.

## Notes

Supplement. **eAppendix.** Diagnosis codes used to define URI and skin conditions subsets **eTable.** Adjusted rate of orders and follow-up visits/events
