# Supplementary material for: Treatment and Follow-up Care Associated With Patient-Scheduled Primary Care Telemedicine and In-Person Visits in a Large Integrated Health System
Source: JAMA Netw Open. 2021 Nov 16;4(11):e2132793. doi: 10.1001/jamanetworkopen.2021.32793 (PMC8596201; doi:10.1001/jamanetworkopen.2021.32793)
Supplement: Supplement. — eAppendix. Diagnosis codes used to define URI and skin conditions subsets eTable. Adjusted rate of orders and follow-up visits/events [file jamanetwopen-e2132793-s001.pdf]

Supplemental Online Content

Reed M, Huang J, Graetz I, Muelly E, Millman A, Lee C. Treatment and follow-up care associated with patient-scheduled primary care telemedicine and in-person visits in a large integrated health system. *JAMA Netw Open.* 2021;4(11):e2132793. doi:10.1001/jamanetworkopen.2021.32793

eAppendix. Diagnosis codes used to define URI and skin conditions subsets

eTable. Adjusted rate of orders and follow-up visits/events

This supplemental material has been provided by the authors to give readers additional information about their work.

© 2021 Reed M et al. *JAMA Network Open.*

eAppendix. Diagnosis codes used to define URI and skin conditions subsets:

URI: 'URI (UPPER RESPIRATORY INFECTION)','COUGH','ACUTE SINUSITIS', 'ALLERGIC RHINITIS', 'PHARYNGITIS'

Skin: 'RASH','ACNE','DERMATITIS','SKIN LESION','ATOPIC DERMATITIS', 'CELLULITIS'

© 2021 Reed M et al. *JAMA Network Open.*

eTable. Adjusted rate of orders and follow-up visits/events

	All visits			Visits with skin dx			Visits with URI-related dx		
	rate	95% CI		rate	95% CI		rate	95% CI	
Any medication									
in-person visit	51.89%	51.82%	51.97%	61.30%	60.99%	61.61%	65.75%	65.55%	65.94%
phone visit	34.70%	34.52%	34.87%	30.59%	29.61%	31.56%	51.19%	50.70%	51.68%
video visit	38.63%	37.97%	39.30%	41.79%	39.98%	43.60%	52.63%	50.62%	54.64%
Antibiotics									
in-person visit	13.45%	13.40%	13.50%	20.43%	20.17%	20.69%	29.36%	29.16%	29.57%
phone visit	9.65%	9.54%	9.76%	14.14%	13.41%	14.87%	25.25%	24.82%	25.68%
video visit	10.63%	10.24%	11.02%	18.01%	16.67%	19.36%	25.55%	23.66%	27.43%
Non-medication order									
in-person visit	59.34%	59.27%	59.41%	47.37%	47.06%	47.69%	46.45%	46.23%	46.68%
phone visit	27.30%	27.14%	27.47%	7.94%	7.33%	8.54%	16.41%	16.02%	16.81%
video visit	29.16%	28.52%	29.80%	11.11%	9.82%	12.40%	17.30%	15.59%	19.02%
In-person visit									
in-person visit	24.51%	24.45%	24.57%	29.50%	29.21%	29.78%	13.59%	13.43%	13.74%
phone visit	26.02%	25.85%	26.19%	38.76%	37.71%	39.81%	23.59%	23.12%	24.06%
video visit	25.37%	24.74%	25.99%	31.28%	29.46%	33.10%	23.24%	21.29%	25.18%
ED									
in-person visit	1.30%	1.29%	1.32%	0.79%	0.73%	0.84%	1.12%	1.07%	1.17%
phone visit	1.37%	1.33%	1.41%	0.81%	0.61%	1.00%	1.25%	1.13%	1.37%
video visit	1.23%	1.06%	1.40%	0.59%	0.27%	0.91%	1.43%	0.85%	2.01%
Hospitalization									
in-person visit	0.23%	0.22%	0.24%	0.13%	0.11%	0.15%	0.16%	0.14%	0.18%
phone visit	0.22%	0.21%	0.24%	0.07%	0.01%	0.14%	0.16%	0.12%	0.21%
video visit	0.23%	0.14%	0.32%	0.13%	-0.04%	0.30%	0.29%	-0.04%	0.61%

Dx=diagnosis; ED=Emergency Department. Adjusted rates are calculated based on results from logistic regression for each outcome.

© 2021 Reed M et al. *JAMA Network Open.*
